# Presentation and Management of a Giant Coronary Artery Aneurysm with a Fistula to the Right Ventricle

**DOI:** 10.1155/2022/7700086

**Published:** 2022-04-28

**Authors:** Nicholas Peterman, Bradley Kaptur, Naveed Adoni

**Affiliations:** ^1^Carle Illinois College of Medicine, University of Illinois Urbana-Champaign, Champaign, IL, USA; ^2^Carle Foundation Hospital, Urbana, IL, USA

## Abstract

A 27-year-old female presented to our emergency department in ventricular tachycardia. During her workup, she was found to have an extremely rare giant aneurysmal left anterior descending artery (LAD) ending in a coronary fistula to the right ventricle (RV). After stabilization, a variety of treatment options were considered, as there is no standard first-line treatment.

## 1. Introduction

A coronary artery fistula (CAF) is a rare finding that is characterized by an abnormal termination of a coronary artery into a cardiac chamber, coronary sinus, pulmonary artery, or pulmonary vein [1]. CAF is predominantly congenital and accounts for 0.4% of cardiac anomalies [1]. It is believed to be present in 0.002% of the total population and has no correlation with either race or sex [1]. CAF causes blood flow to bypass myocardial capillary networks and can subsequently lead to ischemia of the tissue distal to the malformation.

Subsequent compensation leads to progressive dilation of the proximal coronary artery [2]. Clinically significant complications of this interplay of ischemia and compensation are present in 11% of those younger than 20 and in 35% of those older than 20 [2]. Potential sequelae include atherosclerotic deposition, mural thrombosis, and rupture [2]. However, the most common complication is coronary artery aneurysm (CAA) formation, which is defined as the dilation of an arterial segment over 1.5 times that of normal [1]. In rarer cases, the CAA can be classified as a giant coronary artery aneurysm (GCAA) when its diameter is over 20 mm [3]. The overall prevalence of a GCAA in the population is 0.02–0.2%, with other potential causes including Takayasu arteritis, Kawasaki disease, and atherosclerosis [4]. Although GCAA is normally asymptomatic, the concomitant CAF that leads to its creation is not [5]. Reported complications include exertional dyspnea (60%), endocarditis (20%), and angina (3-7%) [1]. Eventual congestive heart failure is seen in nearly a fifth of older patients [1].

## 2. Case Presentation

A 27-year-old female presented to our emergency department with a chief complaint of ongoing chest pain that had persisted for the past 5 days. She also mentioned the onset of extreme lightheadedness, and she believed that she came close to passing out several times. Her chest pain was located at her left chest, between her shoulder blades, and with radiation to her left arm. She denied nausea, vomiting, or diaphoresis, but she noted mild shortness of breath. Her initial blood pressure was 114/78, with a temperature 98.4 degrees Fahrenheit.

Upon interview and outside chart review, it was found that the patient had a significant history of perimembranous ventricular septal defect (spontaneously closed), double-chamber right ventricle, left anterior descending to right ventricle coronary cameral fistula, right ventricular clot (managed with rivaroxaban), and ventricular tachycardia (managed with amiodarone, mexiletine, and metoprolol).

**Figure 1:**
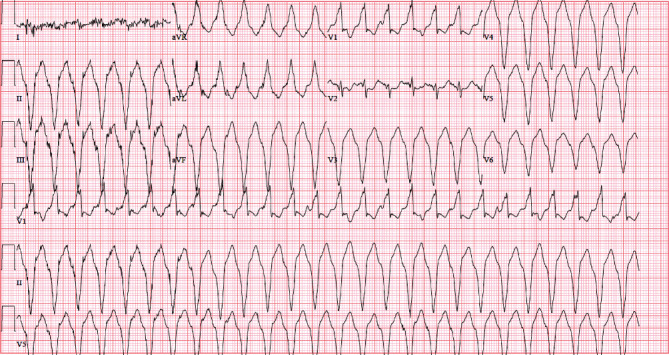
Initial EKG, before cardioversion, showing SMVT. 159 BPM, PR interval 38 ms, QRS duration 188 ms, QT/QTc 322/523 ms.

Sustained monomorphic ventricular tachycardia (SMVT) was noted on her initial EKG (Figure 1), and soon after, the patient appeared diaphoretic and ill with increasing complaints of lightheadedness and palpitations. After initial presentation and identification of VT with precipitous systolic blood pressure drop to the 80s, an IV line was started and etomidate 10 mg IV was given.

**Figure 2:**
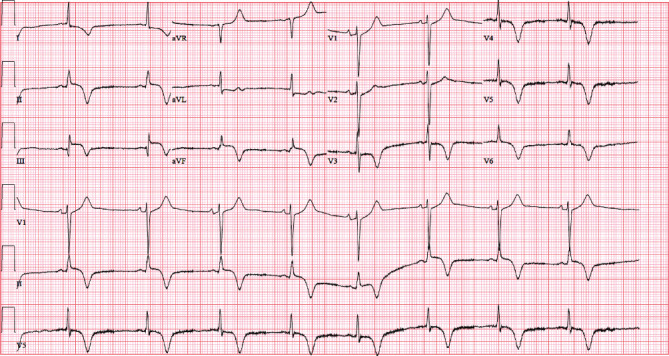
EKG status post cardioversion demonstrating ST elevations in II, III, and AVF. T-wave inversions noted in V3, V4, V5, and V6. 52 BPM, PR interval 106 ms, QRS duration 80 ms, QT/QTc 458/425 ms.

After a lack of improvement, the decision was made to cardiovert. She was cardioverted with 200 joules. Normal sinus rhythm was achieved after cardioversion, and the patient's blood pressure returned to the levels seen on presentation. An EKG postcardioversion was conducted that demonstrated likely inferior STEMI (Figure 2). Her ST elevations status postcardioversion pointed to inferior STEMI as a result of embolus from turbulent flow or reduced cardiac output from SMVT on top of CAD.

At this point, her troponins had climbed from 0.30 to 0.84 within 2 hours of admission, and the patient was brought to the catheterization lab.

**Figure 3:**
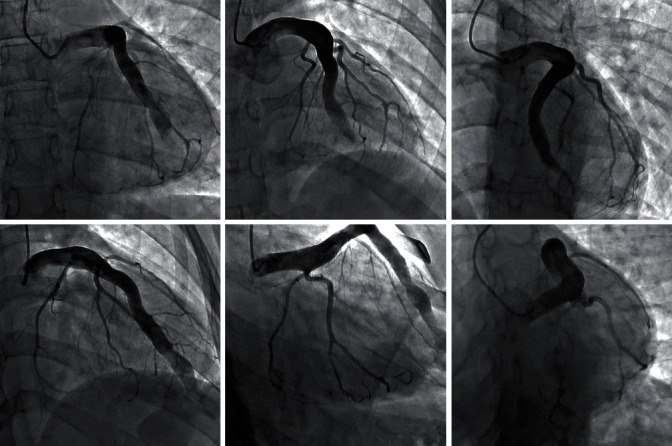
The left main and LAD arteries are diffusely dilated and tortuous, ending in a coronary aneurysm with a fistulous connection to the right ventricular apex. The left anterior descending artery measures 13 mm proximally with a giant aneurysm measuring 21 mm as it fistulizes to the RV apex.

Left heart catheterization (Figure 3) showed the left main, and LAD arteries were diffusely dilated and tortuous, ending in a coronary aneurysm with a fistulous connection to the right ventricular apex. The connection point was separated from the remainder of the chamber by a fibrous, C-shaped band of tissue with minor communication in the apical and upper portions of the right ventricle.

No blockage was identified, and PCI was not conducted. It was believed her VT and elevated troponins were secondary to her structural heart disease, and she was transferred to an outside hospital for surgery.

Ablation options were considered, including traditional catheter ablation, noninvasive ablation, and surgical ablation. The traditional catheter ablation was initially favored by the managing team, but the patient was against the procedure due to high-risk stratification and preference to be managed pharmacologically. A single lead ICD was ultimately implanted subcutaneously given the anatomic constraints of her apical RV and the preference of the patient. The patient was discharged without complications on sotalol, mexiletine, rivaroxaban, aspirin, and atorvastatin.

One month later, the patient had a subsequent episode of VT with ED admission. She was cardioverted to sinus rhythm and monitored for several hours. Her sotalol was changed to amiodarone, and the VT zone settings of her ICD were reprogrammed to lower the threshold for firing. Additionally, the plan to perform outpatient ablation within the next few weeks was discussed with the patient.

After discharge from the emergency department, the patient declined to schedule her ablation procedure, though she did continue with her medication regimen. She was transitioned to sotalol and mexiletine for longer-term VT suppression. Over the subsequent 18 months, she had 1 further episode of sustained tachycardic palpitations with a heart rate in the 150 s. According to the patient, this episode coincided with significant stress in her personal life. At that time, EKG demonstrated MMVT that was below the ICD therapeutic settings. Device interrogation revealed multiple episodes of antitachycardia pacing initiation preceding this episode. She was cardioverted in the ED, and her device settings were once again reprogrammed. Throughout this time, the patient has continued to decline the ablation procedure and has elected to continue with medical management.

## 3. Discussion

Clinical presentation of CAF depends on the magnitude of left-to-right shunting and the possibility of developing coronary steal syndrome [1]. Coronary steal describes the shunting of high-pressure coronary vasculature into the low-resistance circulation through the fistula. The change in pressure between the linked cavities is large enough to precipitate a high volume of flow that “steals” blood flow from the surrounding myocardium and amplifies ischemia. This is especially prominent during exercise [1].

Although rare, concomitant CAF and CAA have been treated surgically before [6]. The repair is done by closing the fistula and then forming a bypass route using a vein graft in order to supply the distal vasculature [6, 7]. However, due to both the giant nature of the aneurysm and the diffuse ectasia of the entire LAD in this patient, the best treatment plan becomes more difficult to determine [6].

Beyond the normal hemodynamics of the fistula, this patient presented with recurrent SMVT originating from the malformation that predisposed her to sudden cardiac death [8]. Along with ablation, an ICD and antiarrhythmic pharmacotherapy would be the standard of care due to VT with structural heart abnormality [1, 8]. Options for implantation are complicated due to her double right ventricle. The fibrous band bisecting her right atrium would obstruct optimal lead placement during transvenous insertion, thus leaving subcutaneous lead placement as the preferred route [9]. The natural history of aneurysms is progressive enlargement. The likely long-term management of this disease process involves intervention [10–13]. However, our patient specifically and adamantly declined catheter ablation of her VT. Thus, in the context of strong patient preference, the ICD was chosen for secondary prevention.

Regarding the choices of medication in our patient, the brief choice of amiodarone was made on the basis of the patient's history, recent AHA and NASPE guidelines, and her QRS and QT intervals. At that time, the potential adverse effects in terms of toxicity involving multiple organ systems were balanced by its superior prevention of sustained ventricular tachyarrhythmias. However, once the patient again declined long-term management in the form of ablation, medication choice in the form of sotalol and mexiletine was resumed.

The mechanism of ST elevation in this patient is another area of interest. In our patient, the EKG postcardioversion demonstrated ST elevations and she had climbing troponins. Per STEMI protocol, she was taken for left heart catheterization. At that time, concerns were high for possible embolus from turbulent follow or reduced cardiac output from SMVT on top of CAD. During the catheterization, no blockage was identified, and thus, PCI was not conducted. At the time, it was believed that her VT and elevated troponins were secondary to her structural heart disease. Coronary steal is a plausible mechanism of ST elevation in this clinical context. In retrospect, the use of fractional flow reserve (FFR) as a diagnostic tool during the initial angiography may have provided additional insight into the responsible mechanism. In the literature, coronary artery aneurysms have been previously associated with unstable angina and STEMI [14–17].

## 4. Conclusions

Ventricular tachycardia can present in the setting of rare structural abnormalities like CAF with GCAA. Subsequent workup requires an understanding of both the unique pathophysiology of the abnormality and an adaptation of related best practice treatments to fit the unique needs of the patient.

## Data Availability

No data were used to support this study.
